# EQ-5D: a plea for accurate nomenclature

**DOI:** 10.1186/s41687-020-00222-9

**Published:** 2020-07-03

**Authors:** Richard Brooks, Kristina S. Boye, Bernhard Slaap

**Affiliations:** 1 EuroQol Group Research Foundation, Marten Meesweg 107, 3068 AV Rotterdam, The Netherlands; 2 Eli Lilly and company, Lilly Corporate Center, DC 893, Indianapolis, IN 46285 USA

**Keywords:** EQ-5D, Nomenclature, EuroQol

## Abstract

Between 1987 and 1990, the EuroQol Group developed a 5-dimension health-related quality of life instrument, originally known as ‘the EuroQol instrument’, which since 1995 has been called the ‘EQ-5D’. For several years, ‘the EuroQol instrument’ and ‘EQ-5D’ were both deployed in published materials. In order to standardise nomenclature, the EuroQol Group agreed in 2001 on a terminology glossary containing 12 items; this was recently revised and augmented to include 22 items and can be found on the Group’s website (www.euroqol.org). Since 2009, EQ-5D has been available in three versions: EQ-5D-3L, EQ-5D-5L, and EQ-5D-Y, where 3L stands for three levels, 5L for five levels, and Y for youth. Yet, almost 20 years after the original glossary was published, the instrument and its components continue to be inaccurately named in published materials. Two surveys – of arthritis applications, and 82 recent publications – found a variety of terms used to describe the instrument. Despite the instrument being named ‘EQ-5D’ for 25 years, and the terms ‘EQ-5D-3L’ and ‘EQ-5D-5L’ being established for a decade, variations of ‘the EuroQol instrument’ continue to be used as descriptors. The EuroQol Group’s website contains advice on how to use EQ-5D, including nomenclature, and potential users are urged to consult the site. Since standardising nomenclature is crucial in the compilation of systematic reviews, the EuroQol Group would like to emphasise that ‘EQ-5D’ is not an abbreviation and is the correct term to use when referring to the instrument in general. In the interests of accuracy and good practice, users of the EuroQol family of instruments should employ the standard EQ-5D nomenclature when reporting and discussing their findings.

## Background

Between 1987 and 1990 the EuroQol Group, a small number of researchers with differing professional backgrounds from the United Kingdom, the Netherlands, Sweden, Finland, and Norway developed a health-related quality of life instrument [1]. Known as ‘the EuroQol instrument’, this was a 6-dimension measure that was further refined to 5 dimensions by 1991. In 1994, it was proposed that ‘EQ5D’ be added to the Group’s logo; this was modified to ‘EQ-5D’, a term that began to be used in papers presented at the Group’s annual Plenary meeting in 1995.

In the years that followed, ‘the EuroQol instrument’ and ‘EQ-5D’ were both deployed in published materials. Early users in clinical and medical settings had become accustomed to the former term; and the Group’s state-of-play paper, published in 1996 [2], had referred to ‘the EuroQol instrument’, so researchers often followed the same convention.

Recognising the need to standardise nomenclature usage for both EQ-5D and its components, in 2001 the EuroQol Group agreed on a terminology glossary containing 12 items. ‘EQ-5D’ was used consistently throughout the book produced for the Biomed EQ-Net project of 1998–2001 [3]. This project also provided definitions of EQ-5D concepts and translation guidelines. The glossary was recently revised and augmented to include 22 items and can be found on the Group’s website (www.euroqol.org)**,** under the heading *Terminology* [4].

Since 2009, EQ-5D has been available in three versions: the three-level EQ-5D-3L; the five-level EQ-5D-5L; and a ‘youth’ adaptation, EQ-5D-Y. Although the EQ is in recognition of the EuroQol group name, the D refers to dimensions, the L signifies level, and the Y stands for youth, the name of the instrument should not be spelled out in this manner as this is inaccurate. Therefore, EQ-5D is not an abbreviation and is the correct term to use when referring to the instrument in general.

Yet, two decades since the glossary was made available, the instrument and its components continue to be inaccurately named in published materials. This is important, not least for researchers undertaking systematic reviews.

Evidence of this initially came from a sample survey of EQ-5D applications in the clinical area of arthritis covering 2012–2017. It became evident in conducting this survey that there were a considerable number of inconsistencies and errors in the representation of EQ-5D. To cover more recent citations of EQ-5D two further samples were taken, comprising a total of 82 papers.

### Arthritis applications

We found a variety of terminology used to describe the instrument; and based on published abstracts, these terms have found their way into the literature.

Notably, in reviewed abstracts the instrument was described variously as a weighted index, an index (score), and an index (scale). This creates considerable potential for confusion because EQ-5D is an instrument for both the measurement and the valuation of health-related quality of life. To be specific: when a respondent completes EQ-5D an EQ-5D profile is obtained, which is a description of this person’s current self-reported health state (e.g. 12211), and an EQ VAS score, which is a score between 0 and 100 recording the person’s overall health-related quality of life. An EQ-5D value, also sometimes referred to as an index, score or utility is the value attached to an EQ-5D state according to a particular EQ-5D value set.

Despite the term ‘EQ-5D’ having been established 25 years ago, and the terms ‘EQ-5D-3L’ and ‘EQ-5D-5L’ being in regular use for a decade, this arthritis literature continued to refer to ‘the EuroQol instrument’ and other variations, such as: EuroQol Health-Related Quality of Life Survey, EuroQol (EQ)-5D, EuroQol-5 dimension, EuroQol questionnaire, and EUROQOL. We also found the ‘EQ-5D’ referred to variously as: ‘EQ5D’, ‘EQ5d’, ‘EQ5-D’, ‘EQ-5D-3L’, ‘EQ5D-5L’, and ‘Eq5D-5L’.

### Two samples of recent citations

1. 20 publications reviewed

Google Scholar was used to check publications citing the EuroQol Group’s 1996 paper [2] and identified 289 citations for 2019, covering a wide range of EQ-5D usage.

We checked the first 20 abstracts (and the full paper, if available), and found that half the papers did not mention EQ-5D in the abstract. Among the 10 articles that did, they referred to it variously as: EuroQol, EQ-5D, EuroQol five-dimension scale questionnaire, EuroQol-5 Dimensions-3 Level (EQ-5D-3L) Scores, and EuroQol-5D (EQ-5D) utility score.

(2) 62 publications reviewed

PubMed was checked for citations of Devlin and Brooks [5] in the current year 2020. Many of the terms listed in the previous paragraph were also in evidence in this sample. A spacing oddity appeared in 7 papers, viz. EQ-5D-3 L and EQ-5D-5 L, including 2 papers containing sections headed EQ-5D-3 l and EQ-5D-5 l. An issue arose with respect to abbreviations lists, which some journals required. This was the case for 19 of the 62 papers and produced such ‘definitions’ of EQ-5D as: EuroQol-5 Dimension, EuroQol 5 Dimensions, European Quality of Life Five Dimension, and EuroQol five-dimensional. There were similar representations for EQ-5D-3L and EQ-5D-5L with the ‘three levels’ and ‘five levels’ spelled out. One paper even had EQ-5D-5L represented as ‘EuroQol 5D-5L’. It is not necessary to ‘spell out’ EQ-5D in these ways and indeed 6 of these papers did not include EQ-5D in their glossaries, as we would expect.

## Discussion

That these limited samples identified such a variety of usage illustrates the problem: the terminology used for the EQ-5D and its components, despite standardisation many years ago, has been inconsistently applied in journal articles.

There is a rigid tradition, in the medical literature, of expanding abbreviations at first mention, especially for health status and health-related quality of life questionnaires and instruments. Some journals evidently require glossaries of abbreviated terms, as indicated above. Because ‘EQ-5D’ looks like an abbreviation, writers may not appreciate that it is simply the name of the instrument and requires no further definition. The same consideration applies to EQ-5D-3L, EQ-5D-3L, and EQ-5D-Y. Journal copy editors or reviewers may add to the confusion by asking for the full name to be spelt out. In complying with this request, as shown above, authors may propose ‘EuroQol-5 Dimension’ or some similar term – perhaps based on the way it was mentioned in articles previously published in that journal.

Given the importance of standard terminology, especially when compiling systematic reviews, we hope this brief Commentary clarifies the correct nomenclature regarding the EQ-5D. We urge researchers and other interested parties using the EQ-5D to consult the Group’s website, which contains clear advice on how to use the EQ-5D. The standard nomenclature is laid out in the form of a 22-term glossary [4]. The detailed *User Guides*, also on the website, are regularly updated to include, for example, information on newly translated versions, and on additional country valuation sets. In addition, there are boxed example texts in the *User Guides* for ‘describing the EQ-5D and reporting and analysing EQ-5D data for study protocols/ proposals’.

## Conclusion

‘EQ-5D’ is not an abbreviation; it is the correct term to use, as are ‘EQ-5D-3L’, ‘ED-5D-5L’, and ‘EQ-5D-Y’ and they require no further definition. In the interests of accuracy and good practice, the EuroQol Group urges users of its instruments to employ the standard EQ-5D nomenclature when reporting and discussing their findings.

## Data Availability

Not applicable.
